# Increased Perfluorooctanesulfonate (PFOS) Toxicity and Accumulation Is Associated with Perturbed Prostaglandin Metabolism and Increased Organic Anion Transport Protein (OATP) Expression

**DOI:** 10.3390/toxics12020106

**Published:** 2024-01-26

**Authors:** Lanie A. Williams, Matthew C. Hamilton, Matthew L. Edin, Fred B. Lih, Jazmine A. Eccles-Miller, Nishanth Tharayil, Elizabeth Leonard, William S. Baldwin

**Affiliations:** 1 Biological Sciences, Clemson University, Clemson, SC 29634, USA; alanna6@clemson.edu (L.A.W.); mchamilton95@gmail.com (M.C.H.); eccles@clemson.edu (J.A.E.-M.); 2 Division of Intramural Research, National Institute of Environmental Health Sciences, National Institute of Health, Research Triangle Park, Washington, NC 27709, USA; matthew.edin@nih.gov (M.L.E.); lih@niehs.nih.gov (F.B.L.); 3 Plant and Environmental Sciences, Clemson University, Clemson, SC 29634, USA; ntharay@clemson.edu (N.T.); eleona2@clemson.edu (E.L.)

**Keywords:** bioaccumulation, PFAS, OATP, transporters, oxylipins, prostaglandins, CYP2B

## Abstract

Perfluorooctanesulfonate (PFOS) is a widespread environmental pollutant with a long half-life and clearly negative outcomes on metabolic diseases such as fatty liver disease and diabetes. Male and female Cyp2b-null and humanized CYP2B6-transgenic (hCYP2B6-Tg) mice were treated with 0, 1, or 10 mg/kg/day PFOS for 21 days, and surprisingly it was found that PFOS was retained at greater concentrations in the serum and liver of hCYP2B6-Tg mice than those of Cyp2b-null mice, with greater differences in the females. Thus, Cyp2b-null and hCYP2B6-Tg mice provide new models for investigating individual mechanisms for PFOS bioaccumulation and toxicity. Overt toxicity was greater in hCYP2B6-Tg mice (especially females) as measured by mortality; however, steatosis occurred more readily in Cyp2b-null mice despite the lower PFOS liver concentrations. Targeted lipidomics and transcriptomics from PFOS-treated Cyp2b-null and hCYP2B6-Tg mouse livers were performed and compared to PFOS retention and serum markers of toxicity using PCA. Several oxylipins, including prostaglandins, thromboxanes, and docosahexaenoic acid metabolites, are associated or inversely associated with PFOS toxicity. Both lipidomics and transcriptomics indicate PFOS toxicity is associated with PPAR activity in all models. GO terms associated with reduced steatosis were sexually dimorphic with lipid metabolism and transport increased in females and circadian rhythm associated genes increased in males. However, we cannot rule out that steatosis was initially protective from PFOS toxicity. Moreover, several transporters are associated with increased retention, probably due to increased uptake. The strongest associations are the organic anion transport proteins (*Oatp1a4-6*) genes and a long-chain fatty acid transport protein (*fatp1*), enriched in female hCYP2B6-Tg mice. PFOS uptake was also reduced in cultured murine hepatocytes by OATP inhibitors. The role of OATP1A6 and FATP1 in PFOS transport has not been tested. In summary, Cyp2b-null and hCYP2B6-Tg mice provided unique models for estimating the importance of novel mechanisms in PFOS retention and toxicity.

## 1. Introduction

Per- and polyfluoroalkyl substances (PFASs) are high-use, slow degrading chemicals used in coatings, paints, stain repellents, fire-fighting foams, and other industrial processes. Their resistance to degradation makes them pervasive and persistent “forever chemicals” [1]. For example, PFOS, a legacy PFAS, has a half-life of about 5.5 years in humans because it binds to lipid binding proteins with a high affinity for saturated fats due to PFOS’s similar structure to fatty acids, with the primary difference being its replacement of hydrogen with fluorine [2,3]. PFASs are measured in the serum of more than 98% of Americans [2,4], with food and water the primary sources of contamination [1,5]. Some common long chain fluorosurfactants such as PFOS and PFOA were phased out of production in the USA and Europe because of their long half-lives, with a few exemptions. However, PFOS has continued to cause adverse health effects due to its bioaccumulation [5,6]. 

PFOS and other PFASs are associated with cardiovascular disease, liver disease, and metabolic diseases such as diabetes, most likely because they effect energy metabolism through the mitochondria [1]. Recent analyses indicates that PFOS, PFOA, PFNA, and several other PFASs are associated with increased liver toxicity biomarkers in blood, such as alanine aminotransferase (ALT), hypercholesterolemia, and high LDL in humans [7,8,9]. The most sensitive tissue to PFOS appears to be liver, with several studies revolving around PFOS’s disruption of normal liver metabolic functions and increase in fatty liver diseases. The liver, and to a lesser extent serum, is where most PFOS bioaccumulates because of the presence of albumin and other fatty acid binding proteins (FABP) [2,3].

Previous work in our laboratory showed that female humanized CYP2B6-transgenic (hCYP2B6-Tg) mice were more sensitive to PFOS than female Cyp2b-null mice as measured by mortality, probably due to increased serum and liver PFOS retention. However, male mice did not demonstrate increased toxicity in the humanized mice compared to the Cyp2b-null mice, despite greater bioaccumulation in mice provided a normal chow diet [10]. There are few mouse models known that can inform toxicologists of novel mechanisms involved in PFOS retention. Therefore, these two mouse models provide unique systems for determining yet-unknown transporters, binding proteins, or other genes and processes involved in PFOS retention. In addition, their differences may also provide information on markers of toxicity. 

CYP2B6 is a cytochrome P450 enzyme that is primarily expressed in the liver and metabolizes many different chemicals, including xenobiotics such as various pesticides, plasticizers, and several pharmaceuticals, steroids such as testosterone and estradiol, and polyunsaturated fatty acids such as linoleic acid (LA), arachidonic acid (AA), and docosahexaenoic acid (DHA) [11,12,13,14,15]. Several PUFA metabolites (oxylipins) are associated with high-fat diet-induced steatosis in hCYP2B6-Tg mice, including 14,15-EET, PGF2a, 9-HODE, and 13-KODE, and to a lesser extent, 11,12-EET, 14,15-DHET, and 9,10-DiHOME. Most high-fat diets are high in n-6 PUFAs and therefore only n-6 oxylipins were captured during this previous in vivo study [11]. Some lipid metabolites may prove to be reasonable biomarkers of PFOS-mediated steatosis or non-alcoholic fatty liver disease (NAFLD). Recent studies indicate that oxylipins may prove to be biomarkers of age, Alzheimer’s, cardiovascular disease, inflammation, and toxicant exposure such as dioxin and PFAS [12,16,17,18,19]. 

The purpose of this study is to (1) test whether there are specific oxylipins associated with PFOS toxicity that may help explain or provide markers of differential toxicity and steatosis between normal diet-fed Cyp2b-null and hCYP2B6-Tg mice, (2) to estimate the mechanism of PFOS toxicity on the liver as tested by transcriptomics, and (3) to determine parameters that are associated with and likely to increase retention in hCYP2B6-Tg mice in comparison to Cyp2b-null mice. Several serum and liver parameters such as ALT, AST, L-FABP, and PFOS concentrations were compared to lipidomic (oxylipins) and transcriptomic (RNAseq) targets through principal component analysis (PCA) to determine potential oxylipins and genes of interest. The results provide new markers of chemically initiated liver disease and provide putative targets for understanding and reducing PFOS uptake and retention. 

## 2. Materials and Methods

### 2.1. PFOS Treatment of Cyp2b9/10/13-null (Cyp2b-null) and Humanized CYP2B6 Transgenic (hCYP2B6-Tg) Mice

Previously, we generated a Cyp2b-null mouse model on a C57Bl/6J (B6) background. Crispr/Cas9 was used to delete the primarily hepatic *Cyp2b* genes, *Cyp2b9*, *Cyp2b10*, and *Cyp2b13* located in tandem repeat on chromosome 7. Mice have two other *Cyp2bs*, but they are not primarily expressed in adult mouse livers and are located six genes away from the *Cyp2b* cluster. To avoid impacting these intervening genes, only *Cyp2b9/10/13* were knocked out, generating a 287 kb deletion [20,21]. Humanized CYP2B6-transgenic mice (hCYP2B6-Tg) were generated on the Cyp2b-null background by breeding CYP2B6/2A13/2F1-transgenic mice [22] with Cyp2b-null mice [11]. DNA isolated from tail clippings of bred mice was genotyped using the AccuStart II Mouse Genotyping Kit from QuantaBio (Beverly, MA, USA) to confirm the *Cyp2b9/10/13* knockout and/or presence of human CYP2B6 prior to PFOS treatment [10,11]. 

Male and female Cyp2b-null and hCYP2B6-Tg mice between 10 and 12 weeks old were administered 0, 1, or 10 mg/kg/day PFOS (0-, 1-, or 10- PFOS) (Sigma-Aldrich, St. Louis, MO, USA) for 21 days via oral gavage (n = 5–7). PFOS was dissolved in a solution of water and 0.5% Tween 20. The mice were fed a normal diet (ND; 2018S-Envigo Teklad Diet, Madison, WI, USA) containing 18% of its kcal as fat [10]. Mice were anesthetized by administration of 3% isoflurane and euthanized via heart puncture/blood collection followed by CO_2_ asphyxiation. Livers were immediately excised, snap frozen in liquid nitrogen, and stored at −80 °C until RNA extraction [10]. All work with mice was approved through Clemson University’s IACUC—Animal Use Protocol AUP2019-061. 

### 2.2. Sample Preparation—RNA Extraction and Serum Analysis

RNA extraction was performed with about 20 mg of liver tissue homogenized in 1 mL TRIzol (Life Technologies, Carlsbad, CA, USA) according to the manufacturer’s protocols. RNA was dissolved in 100 μL RNase-free water, and sample quantity and quality quantified using a NanoDrop (Thermo Fisher Scientific, Waltham, MA, USA), confirmed on a Qubit 4 benchtop fluorometer (Invitrogen, Waltham, MA, USA) and stored at −80 °C. 

Serum was isolated from blood via centrifugation. Different serum parameters were previously measured from these mice [10], including alanine aminotransferase (ALT), aspartate aminotransferase (AST), alkaline phosphatase (ALP), and albumin concentrations using a Beckmann Coulter AU480 analyzer and respective Beckman Coulter kits according to the manufacturer’s protocol (Beckman Coulter, Brea, CA, USA)(n = 3–5). In addition, liver fatty acid binding protein (L-FABP) was measured from serum (n = 2–7) and liver cytosol (n = 2–3) using a sandwich ELISA kit from LifeSpan BioSciences, Inc. (Lynnwood, WA, USA). The ELISA was performed in a 96-well plate according to the manufacturer’s protocol, and the absorbance was read at 450 nm on a Synergy H1 plate reader (BioTek, Winooski, VT, USA). 

### 2.3. Oxylipin Extraction and Quantification

For quantification of free and total liver oxylipins, frozen livers were weighed and placed in a tube with 49 µL HBSS/mg liver. Each tube received 1 µL of 1 mM 1-Trifluoromethoxyphenyl-3-(1-propionylpiperidin-4-yl) urea (TPPU) in methanol. Liver tissue was homogenized using a Tissuelyzer II (Qiagen) set to 30 Hz for 10 min. For free oxylipins, a 10 µL internal standard mix (3 ng PGE2-d9, 3 ng LTB4-d4, 1.5 ng 11,12-DHET-d11, 3 ng 11,12-EET-d11, 3 ng 15-HETE-d8, and 7.5 ng AA-d11, all from Cayman Chemical) was added to 200 µL (4 mg of liver homogenate) and extracted by liquid:liquid extraction with 800 µL ethyl acetate and dried under vacuum centrifugation. All reference standards and internal standards are commercially available from Cayman Chemical Company (Ann Arbor, MI, USA). To determine “Total” oxylipins (free and sn-2 esterified), a separate quantity of 100 µL (2 mg of lysate) was incubated with 3 units of porcine pancreas PLA_2_ (Sigma) to hydrolyze sn-2-esterified oxylipins before identical liquid:liquid extraction. Oxylipins were assayed on an Ultimate 3000 UHPLC equipped with an Xselect CSH C18, 2.1 × 50 mm, 3.5 μm particle column (Waters, Wilford, MA, USA), and a TSQ Quantiva tandem mass spectrometer (Thermo Fisher Scientific, Waltham, MA, USA). Quantification was determined using multiple reaction monitoring and quantified by a blinded observer using TraceFinder (v4.1, ThermoFisher Scientific, Waltham, MA, USA). Recovery of internal standards across all samples (peak area of input vs. peak area measured in samples) was determined: PGE2-d9 (96 +/− 1%), LTB4-d4 (89 +/− 1%), 11,12-DHET-d11 (95 +/− 2%), 11,12-EET-d11 (94 +/− 1%), 15-HETE-d8 (93 +/− 2%), and AA-d11 (100 +/− 3%). Relative response ratios of analytes were compared to standard curves generated with oxylipins purchased from Cayman Chemical (Ann Arbor, MI, USA). Further details on the methods, mass-to-charge ratios, and standard curve generation are available in the Supplementary Materials under Oxylipin Standard Methods.

### 2.4. RNA Sequencing and Analysis

RNA sequencing was performed by Novogene (Sacramento, CA, USA). A cDNA library was prepared from the messenger RNA using poly A enrichment and quantified with a Qubit (≥0.5 ng/μL). Samples were sequenced with a paired-end 150 NovaSeq 6000 system (Illumina, San Diego, CA, USA) to an average of 22,929,441 raw read pairs per sample. Raw read processing was performed using the Palmetto Cluster (Anderson, SC, USA) servers operating on Linux as described previously [11,23] with some updates. Quality checks were performed using FastQC [24]. Trim Galore was used to trim adaptor sequences and then aligned to the reference genome (*Mus musculus*, GCF_000001635.27) using the program RNA-Seq by Expectation-Maximization (RSEM) [25]. Mapped reads were counted with featureCounts [26]. Differential expression analysis from the featureCounts output was performed in R using edgeR. Low-level expression genes were filtered out using the requirement that at least 3 of the 4 samples per group must have greater than 1 count per million. Samples were then normalized within edgeR [27]. Differentially expressed genes were selected based on false discovery rate (FDR < 0.05) and log fold change (logFC < −1 or logFC > 1). Enriched gene ontology (GO) terms were determined separately for the significantly upregulated (logFC > 1) and downregulated (logFC < 1) genes in each comparison, as well as for all the differentially expressed genes together [28]. Significant GO terms (FDR < 0.05) were grouped by semantic similarity and graphically represented by ReviGo [29]. Identification and visualization of KEGG pathways and transcription factors associated with significantly up- and downregulated genes were identified using DAVID [30] and EnrichR [31], respectively.

### 2.5. Hepatocyte PFOS Uptake Inhibition Assay

Murine hepatocytes isolated from ICR/CD-1 female livers from BioIVT (Westbury, NY, USA) were plated on a collagen-coated, 12-well plate with a Matrigel overlay. The cells were cultured in Invitrogro CP Rodent Medium with TORPEDO antibiotic mix (BioIVT) for 24 h at 37 °C prior to the experiment. To inhibit Oatp1a activity, the hepatocytes were exposed to known Organic Anion Transport Protein (Oatp) inhibitors, digoxin (primarily Oatp1a4 inhibitor), naringin (primarily Oatp1a5 inhibitor), and bromsulfthalein (broad Oatp inhibitor). Treatment groups (n = 3–4) included a no-drug treatment control, a 10 μM digoxin treatment, and a 60 μM drug mixture (10 μM digoxin, 20 μM naringin, and 30 μM bromsulfthalein) (Millipore Sigma, St. Louis, MO, USA). All inhibitors and PFOS were dissolved in dimethyl sulfoxide (DMSO), and DMSO levels were adjusted so that they remained at 0.3% in each well. The inhibitors were incubated with the hepatocytes for 30 min in 1 mL cell culture media prior to exposure to 6 μM PFOS for 3 min as described previously [32]. Three background blanks were run: (1) cells plus PFOS with cells scraped away prior to adding lysis buffer to determine if PFOS sticks to the well and inflates PFOS recovery; (2) no cells plus PFOS to determine the amount of PFOS that sticks to the 12-well plates; and (3) no cells and no PFOS added to determine the amount of PFOS extracted from plates and solvents as background PFOS because it is so pervasive. 

The medium with drugs and PFOS was aspirated off, and each well was washed twice with cold PBS. Cells were lysed with 200 μL of 1% Triton-X-100 in H_2_O and incubated for 20 min at room temperature before collection. PFOS was then extracted. A 150 μL aliquot of cell lysate was placed into a separate tube and 1.25 ng/mL of ^13^C4-PFOS was added as an internal standard. To each sample, 1.2 mL of 5% LC-MS/MS-grade ammonium acetate (2 μM) was added to 95% LC-MS/MS-grade acetonitrile and vortexed for 5 s. Samples were centrifuged at 2500× g for 20 min at room temperature and were diluted 1:1 in LC-MS/MS-grade H_2_O for LC-MS/MS analysis by the Multi-User Analytical Lab (MUAL) at Clemson University, Clemson, SC, USA. 

### 2.6. PFOS Quantification by LC-MS/MS

Before extraction, all samples were spiked with the internal standard, ^13^C4-PFOS (Wellington Laboratories, Guelph, ON, Canada, Product code: MPFOS). LC-MS/MS protocols for liver tissue extracts were previously described by us [10]. Briefly, ultra-fast liquid chromatography was performed on tissue PFOS extracts with a SHIMADZU Prominence ultra-fast liquid chromatography system (Columbia, MD, USA). A Waters X-Bridge C18 (100 mm × 4.6 mm, 5 µm, Milford, MA, USA) column was used for chromatographic separation. The solvents used for the mobile phase were 0.1% formic acid in water and 0.1% formic acid in acetonitrile. The following solvent gradient was used: an increase in formic acid/acetonitrile from 70 to 90% over 8 min; the gradient was then returned to original conditions at 8 min. A Quadripole-Trap 4500 with electrospray ionization (ESI) interface (AB Sciex, Framingham, MA, USA) was used for mass spectrometry. Analysis occurred in Multiple Reaction Monitoring (MRM) mode, and the MRM ion pair used was 498.8/79.8.

LC-MS/MS analysis on the PFOS extracts from primary murine hepatocytes was performed on the SCIEX Triple Quadrupole 4500 Liquid Chromatography Tandem Mass Spectrometer (LC-MS/MS) System (SCIEX, Framingham, MA, USA) by the Multi-User Analytical Lab (MUAL) at Clemson University. Methods were modified from those described previously [10,33,34]. Dilutions of the internal standard in acetonitrile at 1, 5, 15, 100, 250, and 500 ng/mL were used to verify PFOS retention time and to generate a standard curve (R^2^ = 0.9979). Ten μL of each sample was injected, and samples that were above the upper limit of quantification were diluted and reanalyzed. Buffers comprising 0.1% ammonium hydroxide in methanol and 0.1% ammonium hydroxide in water were used to run the mobile phase. A Waters X-Bridge C18 (100 mm × 4.6 mm, 5 µm, Milford, MA, USA) column was used for chromatographic separation. The following solvent gradient was used: 40% of 0.1% ammonium hydroxide in methanol from 0.01–0.1 min, 80% at 0.6 min, 100% at 2–3.5 min, and 40% at 3.51 min through the end of the experiment at 6.5 min. Analysis occurred in Multiple Reaction Monitoring (MRM) mode, and the MRM ion pair used was 498.8/79.8 as described above. Data were recorded with Analyst 1.6.3 and analyzed with MultiQuant 3.0.1 (Framingham, MA, USA) software. Statistical analysis between groups was performed on GraphPad Prism 7.0 (LaJolla, CA, USA) with one-way ANOVA for tissue data and Student’s *t*-test for hepatocyte data.

### 2.7. Principle Component Analysis (PCA)

Biplots were generated with raw gene expression count data for both males and females. These PCA plots included all genes that were differentially expressed (FDR < 0.05) between genotypes and/or PFOS concentration in each sex. These genes were correlated with PFOS concentration, and serum parameters including ALT levels, AST levels, albumin, and L-FABP. Similarly, biplots were created with all oxylipin concentrations and correlated with the previously mentioned serum parameters. Separate oxylipin biplots were created with male total, male free, female total, and female free oxylipins. PCA plots were generated using JMP Pro 17.0 (SAS Institute, Charlotte, NC, USA).

## 3. Results

### 3.1. PFOS Concentrations Are Genotype and Sex Regulated

Previous research primarily focused on the role of diet in PFOS-mediated toxicity and steatosis demonstrated that hCYP2B6-Tg mice retained more PFOS in their liver and serum (Table 1) than Cyp2b-null mice in both female and male mice [10]. In addition, sexually dimorphic differences in retention were observed in the Cyp2b-null mice as the females retained significantly lower concentrations of PFOS than the male Cyp2b-null mice (Table 1). This sex difference did not occur in hCYP2B6-Tg mice and, therefore, the genotype differences in PFOS retention are much more prominent in the females. For example, there is a 14% difference in hepatic PFOS retention between Cyp2b-null and hCYP2B6-Tg males, but a 39% difference in hepatic PFOS retention between Cyp2b-null and hCYP2B6-Tg females. Not surprisingly, hCYP2B6-Tg females showed greater acute toxicity and ALT levels than Cyp2b-null females (Supplementary Materials) [10]; however, normal chow-diet-fed hCYP2B6-Tg mice were protected from steatosis relative to Cyp2b-null mice [10]. Therefore, we are using an -omics approach to investigate why hCYP2B6-Tg mice have less steatosis than Cyp2b-null mice while retaining more PFOS (and showing more toxicity in females). These two models provide a unique resource for determining novel genes involved in PFOS retention and transport. 

### 3.2. PFOS Disrupts Hepatic Oxylipin Levels

**Figure 1:**
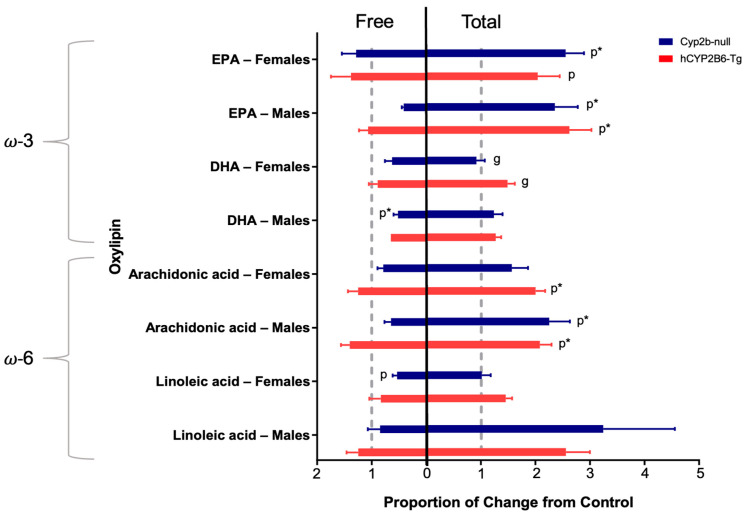
Free and total hepatic oxylipins from Cyp2b-null and hCYP2B6-Tg mice treated with 10 mg/kg/day PFOS. Ratios of targeted oxylipins from 10-PFOS-treated mice to untreated mice. A ratio less than 1 indicates a PFOS-mediated reduction in oxylipins and a ratio greater than 1 indicates a PFOS-mediated increased in oxylypins. Data are presented as the ratio ± SEM. Raw and summary oxylipin data can be found in the Supplemental Materials. Statistical significance was determined via one-way ANOVA followed by Tukey’s post hoc test with GraphPad Prism 7. “g” indicates a difference between genotypes at the same PFOS concentration and “p” indicates a difference from the control caused by PFOS. No asterisk indicates *p* < 0.05, * indicates *p* < 0.01.

Previous research demonstrated that there are several oxylipins produced directly by human CYP2B6 and some of these oxylipins are associated with high-fat diet mediated steatosis, such as 9-HODE, 14,15-EET, PGF2a, and 13-KODE [11,19]. Therefore, we measured hepatic concentrations of free and total (free + membrane bound) LA, DHA, eicosapentaenoic acid (EPA), and AA-derived oxylipins in the PFOS-treated Cyp2b-null and hCYP2B6-Tg mice. Oxylipin species analysis of each PUFA showed that 10-PFOS decreased free DHA-derived oxylipins in the males and free LA-derived oxylipins in the females. Total AA- and EPA-derived oxylipins more than doubled in both males and females treated with 10-PFOS (Figure 1), with the exception of the AA-derived oxylipins in Cyp2b-null females, which saw lesser increases. Most differences in total oxylipins were caused by PFOS, but genotypic differences were observed between DHA-derived oxylipins of females treated with 10-PFOS. Mean ± SEM concentrations of all individual oxylipins can be found in the Supplementary Materials (stored in Mendelay Data) where the sum of derived oxylipins is also graphed. 

PCA was performed on all oxylipins and compared to changes in liver and serum PFOS concentrations, AST, ALT, ALP [10], albumin, and serum and liver L-FABP (Supplementary Materials) to investigate changes associated with PFOS toxicity. 

**Figure 2:**
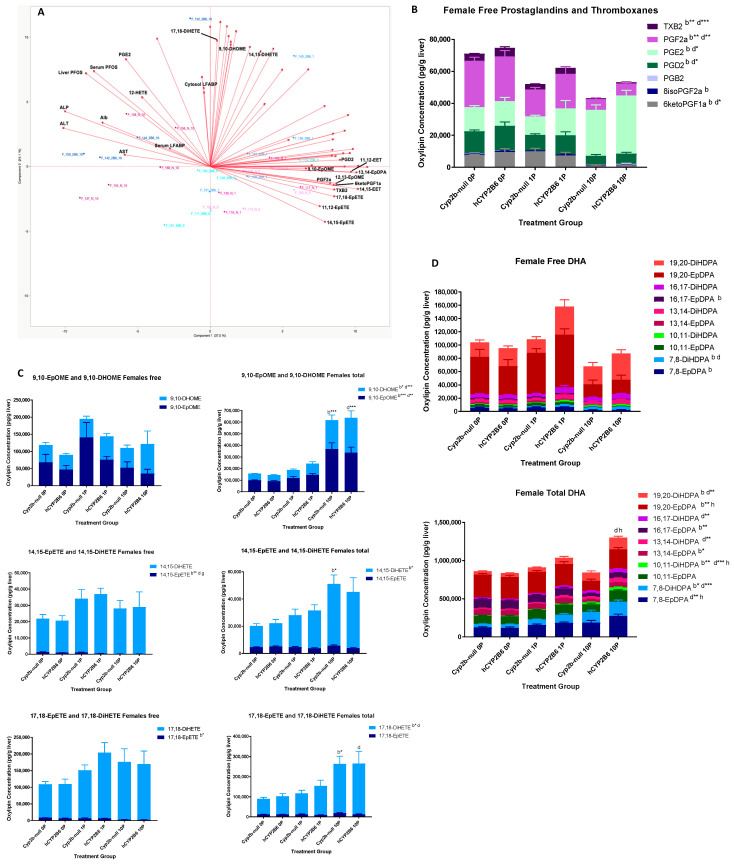
Several oxylipins are associated or inversely associated with PFOS retention and toxicity in females. Principal component analysis (PCA) was performed on measured free oxylipins in comparison to L-FABP, PFOS retention, and serum albumin, ALT, ALP, and AST (**A**). Analysis via two-way ANOVA followed by Tukey’s post hoc test demonstrated that prostaglandins (**B**), epoxide metabolism and membrane release (**C**), and DHA-derived oxylipins (**D**) were perturbed by PFOS treatment. Data in (**B**–**D**) are mean ± SEM with two-way ANOVA followed by Tukey’s post hoc test for multiple comparisons. Significance is shown as follows: “b” demonstrates a difference between untreated and 10 mg/kg/day PFOS in the Cyp2b-null mice, “d” demonstrates a difference between untreated and 10 mg/kg/day PFOS in the hCYP2B6-Tg, “g” demonstrates a difference between the 1 mg/kg/day PFOS-treated Cyp2b-null and hCYP2B6-Tg mice, and “h” demonstrates a difference between the 10 mg/kg/day PFOS-treated Cyp2b-null and hCYP2B6-Tg mice. Differences in total oxylipins are shown above the bars; differences between specific oxylipin species are shown in the legend because there are so many species to evaluate across different treatments and genotypes. Letter by itself indicates *p* < 0.05, * *p* < 0.01, ** *p* < 0.001, *** *p* < 0.0001.

In females, the free oxylipins associated with the retention and toxicity parameters named above include only 12-HETE and PGE2. Oxylipins inversely associated with these same parameters include TBX2, PGF2a, 6-ketoPGF1a, 17,18-EpETE, 14,15-EpETE, 11,12-EpETE, 14,15-EET, 12,13-EpOME, 9,10-EpOME, and 13,14-EpDPA (Figure 2A). We observed from this data that some prostaglandins were repressed (TBX2, PGF2a, 6-ketoPGF1a, PGD2) and PGE2 was significantly increased (Figure 2B). Reductions in CYP-derived oxylipins are consistent with inflammatory conditions [35,36]. All of the other oxylipin metabolites that were inversely associated (reduced in concentration) with increasing PFOS concentrations were epoxides, suggesting that epoxide hydrolase activity was induced by PFOS treatment in the females and, in turn, dihydroxy-oxylipins were produced [12,37]. 

However, to our surprise, the major change in several of these oxylipins was the significant increase in total, rather than free, oxylipin levels (Figure 2C). Last, we investigated DHA oxylipin species because of significant changes in total DHA-derived oxylipin species (Figure 1 and Figure 2D). Differences in female total DHA oxylipin content were primarily caused by increases in 19,20- and 7,8-species following 10-PFOS treatment in the hCYP2B6-Tg mice. 

**Figure 3:**
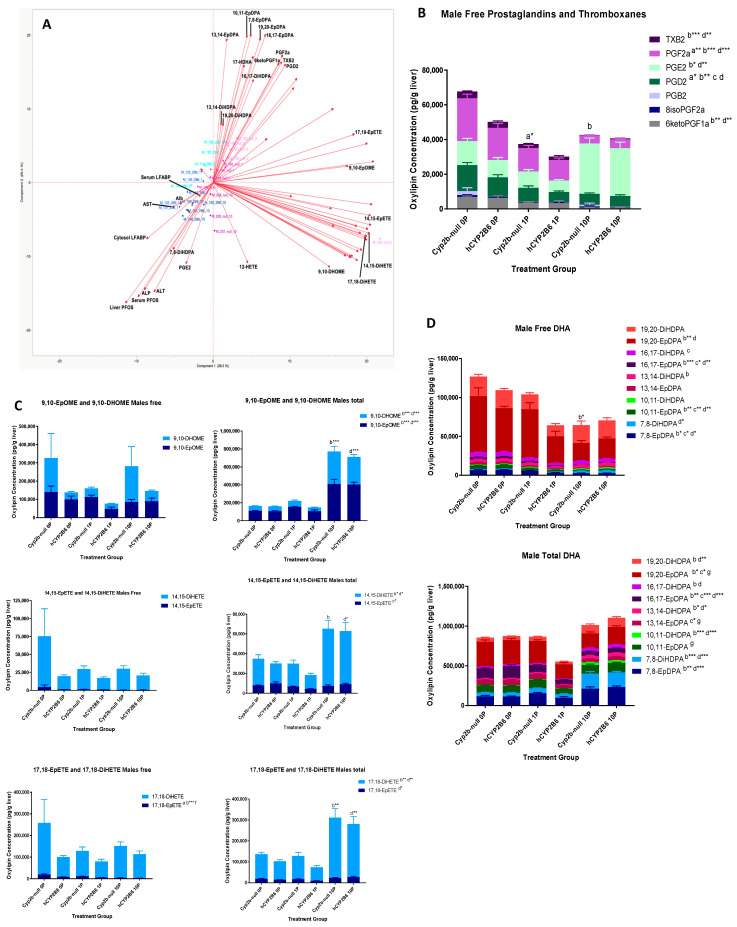
Several oxylipins are associated or inversely associated with PFOS retention and toxicity in males. Principal component analysis (PCA) was performed on measured free oxylipins in comparison to L-FABP, PFOS retention, and serum albumin, ALT, ALP, and AST (**A**). Analysis by two-way ANOVA followed by Tukey’s post hoc test demonstrated that prostaglandins (**B**), some total oxylipins (**C**), and DHA-derived oxylipins (**D**) were perturbed by PFOS treatment. Data in (**B**–**D**) are mean ± SEM and significance is shown as follows: “a” demonstrates a difference between untreated and 1 mg/kg/day PFOS in the Cyp2b-nulls, “b” demonstrates a difference between untreated and 10 mg/kg/day PFOS in the Cyp2b-null mice, “c” demonstrates a difference between untreated and 1 mg/kg/day PFOS in the hCYP2B6-Tg, “d” demonstrates a difference between untreated and 10 mg/kg/day PFOS in the hCYP2B6-Tg, “f” demonstrates a difference between the untreated Cyp2b-null and hCYP2B6-Tg mice, “g” demonstrates a difference between the 1 mg/kg/day PFOS-treated Cyp2b-null and hCYP2B6-Tg mice. Differences in total oxylipins are shown above the bars; differences between specific oxylipin species are shown in the legend because there are so many species to evaluate across different treatments and genotypes. Letter by itself indicates *p* < 0.05, * *p* < 0.01, ** *p* < 0.001, *** *p* < 0.0001.

In males, the free oxylipins strongly associated with serum and liver PFOS concentrations, ALT, ALP, and serum L-FABP include only 7,8-DiHDPA and PGE2 (Figure 3A). 12-HETE, which is associated with PFOS concentrations in females, is only weakly associated in males. PGE2 showed strong association with toxicity and PFOS concentrations in both males and females (Figure 2 and Figure 3). Oxylipins inversely associated with these same parameters include PGF2a, PGD2, 6-ketoPGF1a, TBX2, 7,8-EpDPA, 10,11-EpDPA, 13,14-EpDPA, 16,17-EpDPA, 19,20-EpDPA, 13,14-DiHDPA, 16,17-DiHDPA, 17-HDHA, and 19,20-DiHDPA (Figure 3A). Males, similar to females, also showed repression of prostaglandins, with PGF2a, 6-ketoPGF1a, and TBX2 repressed in both females and males (Figure 3B). A distinct difference between the presence of epoxides and dihydroxy-oxylipins was not observed in the males or was not as pronounced (Figure 3C). There were also genotypic differences. For example, the hCYP2B6-Tg mice showed repression of some oxylipins such as 17,18-EpETE in comparison to Cyp2b-null mice (Figure 3C); a few of the total DHA-derived oxylipins such as 10,11- and 19,20-EpDPA were also lower in the 1-PFOS hCYP2B6-Tg mice compared to the 1-PFOS Cyp2b-null mice (Figure 3D). A large number of free DHA-derived oxylipins were inversely associated with PFOS. Several were differentially expressed (Supplementary Materials), but this may be due in part to reduced conversion from membrane bound to free DHA-derived oxylipins (Figure 3D). 

### 3.3. RNAseq Demonstrates That PFOS Induces Xenobiotic and Lipid Metabolism and Represses Immune Function

RNAseq was performed in the female and male Cyp2b-null and hCYP2B6-Tg mice provided 0, 1, or 10 mg/kg/day PFOS for a total of 48 samples from 12 groups. The data demonstrated a PFOS-mediated dose-dependent increase in the number of genes perturbed relative to the control mice of each genotype and sex (Table 2). The female 10-PFOS-treated hCYP2B6-Tg mice showed the greatest number of gene expression changes, which is not surprising given this group also showed the most acute toxicity [10]. Female and male hCYP2B6-Tg treated with 1-PFOS also showed a greater number of genes significantly altered relative to Cyp2b-null mice. This is consistent with the trend of greater PFOS concentrations in the treated hCYP2B6-Tg mice and inversely associated with previously measured steatosis [10]. 

Few genes were differentially expressed when comparing untreated Cyp2b-null and hCYP2B6-Tg mice. Of great interest because of the differences in toxicity, female mice treated with 10-PFOS showed a relatively large difference in the number of genes expressed between the two genotypes; (235), while males a relatively small difference (18).. Both females and males showed increased PFOS retention in the hCYP2B6-Tg mice compared to the Cyp2b-nulls; however, the female mice had greater retention differences by genotype and greater gene expression differences (Table 1 and Table 2) [10]. 

**Figure 4:**
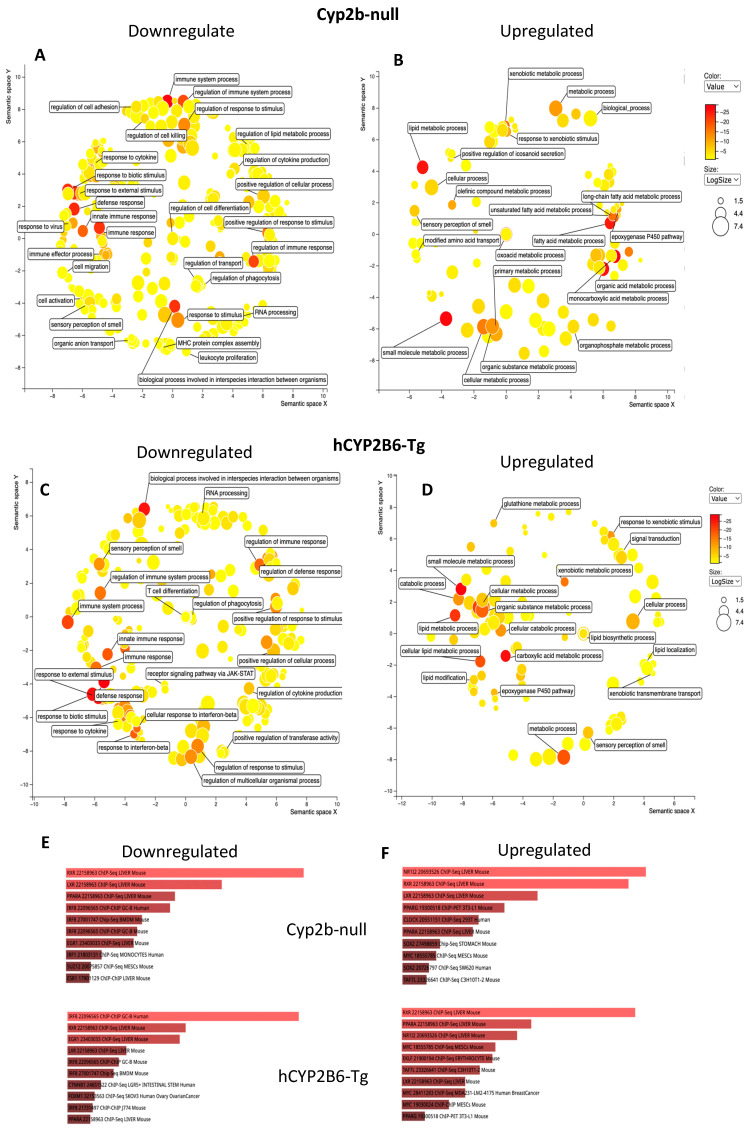
Gene ontology as visualized through Revigo and Enrichr indicates that 10 mg/kg/day PFOS represses immune function and increases lipid uptake, synthesis, and metabolism through nuclear receptors such as RXR, PXR, PPARs, and LXR in female mice. Gene ontologies downregulated (**A**) and upregulated (**B**) by PFOS in hCYP2B6-Tg mice. Gene ontologies downregulated (**C**) and upregulated by PFOS (**D**) in Cyp2b-null mice. Transcription factors putatively involved in the downregulation (**E**) and upregulation (**F**) of these genes by PFOS based on Enrichr. Top (and longer) bars indicate greater confidence in the roll of this transcription factor with red indicating statistical significance; gray not significant (not shown).

**Figure 5:**
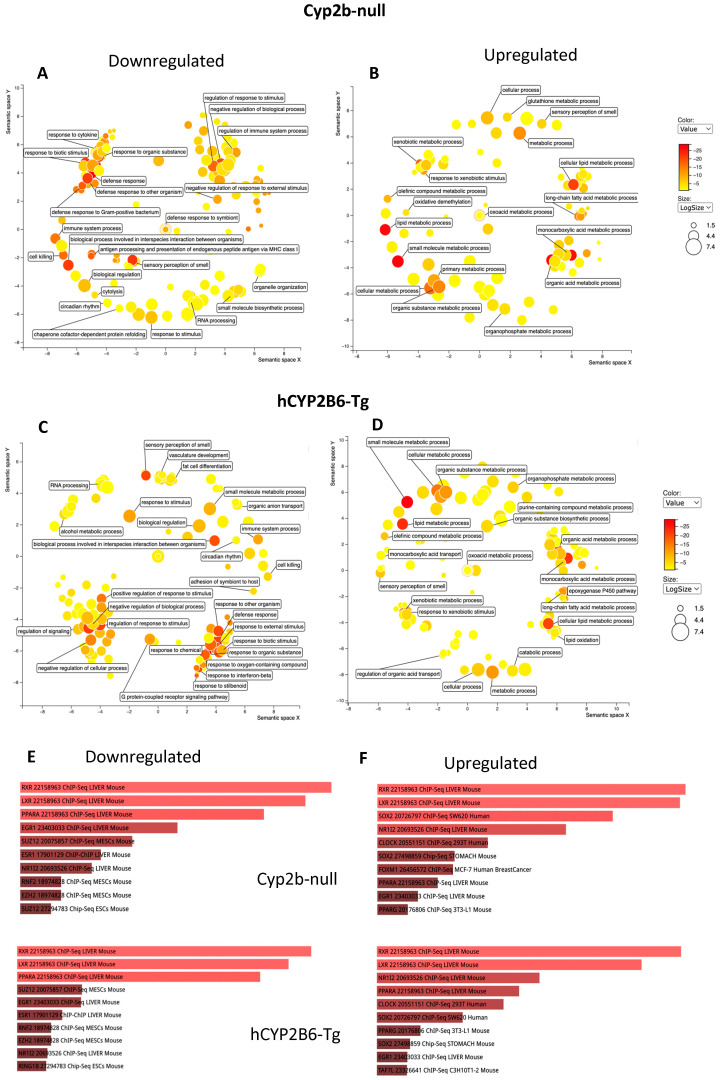
Gene ontology as visualized through Revigo and Enrichr indicates that 10 mg/kg/day PFOS represses immune function and increases lipid uptake, synthesis, and metabolism through nuclear receptors in male mice. Gene ontologies downregulated (**A**) and upregulated (**B**) by PFOS in Cyp2b-null mice. Gene ontologies downregulated (**C**) and upregulated by PFOS (**D**) in hCYP2B6-Tg mice. Transcription factors putatively involved in the downregulation (**E**) and upregulation (**F**) of these genes by PFOS based on Enrichr. Top (and longer) bars indicate greater confidence in the roll of this transcription factor with red indicating statistical significance; gray not significant (not shown).

Gene ontology visualized through Revigo indicates that 10 mg/kg/day PFOS represses the immune response and increases xenobiotic responses, lipid uptake, synthesis, and metabolism genes in females with few major differences between hCYP2B6-Tg and Cyp2b-null mice (Figure 4). Similar GO terms were perturbed in males, except more ontologies related to circadian rhythms and G-protein coupled receptor interactions were downregulated following PFOS treatment (Figure 5). Lists of genes differentially expressed by genotype, sex, or PFOS treatment are provided in the Supplementary Materials. P450 epoxygenases were one of the GO terms altered by PFOS within the lipid metabolism cluster (Figure 4 and Figure 5). Epoxide hydrolase 1 and 2 (*ephx1* and *ephx2*) were induced in females and *ephx1* was induced in males at 10-PFOS consistent with the decreased oxylipin epoxides (Figure 2 and Figure 3). 

EnrichR (Chip-Seq) was performed to determine the transcription factors potentially involved in the regulation of these genes by 10-PFOS. RXR, PXR, PPARα, LXR, and PPARγ are the most likely activated transcription factors in these processes in males and females. There are some other receptors potentially involved, including estrogen and epidermal growth factor. PPARα has often been implicated in PFOS action [38,39,40]. All of these nuclear receptors are involved in lipid homeostasis, including fatty acid, cholesterol/oxysterol, and xenobiotic metabolism [39,41,42,43,44,45,46]. Clock was also found on the list of target transcription factors. At 1-PFOS, several CYPs known as common biomarkers of PXR and PPARα activity (Cyp2-4 families) are high on the list of genes perturbed, indicating their activation at the lower concentration provided [42,47], with a greater number in the hCYP2B6-Tg mice than the Cyp2b-nulls. For example, Cyp3a members were not increased in Cyp2b-null females, but five of the eight Cyp3a members were induced in the hCYP2B6-Tg female mice at 1-PFOS. Similar transcription factors were activated at 1-PFOS, as determined by EnrichR, although fewer genes differentially expressed in the 1-PFOS treated mice than the 10-PFOS treated mice also meant fewer ontologies perturbed (data in Supplementary Materials). However, 1-PFOS did have a profound effect on lipid metabolism (upregulation), with lesser effects on xenobiotic metabolism, circadian rhythms, apoptosis, and development. 

**Figure 6:**
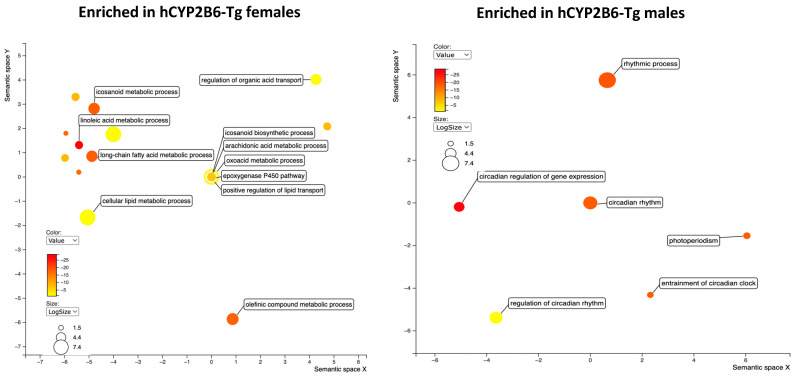
Gene ontology as visualized through Revigo indicates that hCYP2B6-Tg mice treated with 1 mg/kg/day PFOS are enriched for lipid metabolism and lipid/organic anion transport processes in females and circadian rhythm genes in males in comparison to 1 mg/kg/day PFOS-treated Cyp2b-null mice.

Last, because Cyp2b-null mice showed greater steatosis than hCYP2B6-Tg mice treated with 1-PFOS in both females and males, we compared GO terms between the two models. No GO terms were significantly enriched in the Cyp2b-null mice despite several genes being enriched. GO terms were significantly enriched in both the female and male hCYP2B6-Tg mice, with females showing increases in lipid metabolism, organic acid transport, and lipid transport, and males showing changes in circadian rhythms (Figure 6). Increased lipid metabolism in the females included dose-dependent induction in P450 epoxygenases and may provide insight into decreased EpETE and increased EpDPA metabolites that could prove to be protective [37]. Furthermore, the more robust transcriptional response (more genes responding), which includes enhanced induction of CYPs and other genes involved in circadian rhythms, lipid metabolism, and lipid transport, is associated with protection from steatosis observed in the hCYP2B6-Tg mice provided 1-PFOS in comparison to the Cyp2b-null mice provided 1-PFOS. 

### 3.4. Cyp2b-Null Mice Express Significantly Lower PFOS Transporting OATPs than hCYP2B6-Tg Mice

**Figure 7:**
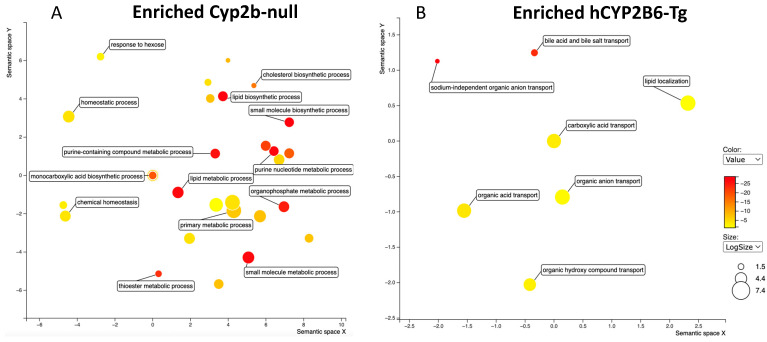
Gene ontology differences between Cyp2b-null (**A**) and hCYP2B6-Tg (**B**) female mice provided 10 mg/kg/day PFOS as visualized through Revigo.

GO analysis via Revigo comparing 10-PFOS-treated Cyp2b-null and hCYP2B6-Tg mice showed no significant differences in males (not shown; Supplementary Files). GO analysis via Revigo showed that hCYP2B6-Tg female mice were enriched with genes involved in organic acid transport, sodium-independent organic acid transporters, bile acid and bile salt transporters, and lipid localization similar to the 1-PFOS-treated mice (Figure 7). Terms enriched in the Cyp2b-null mice include thioester metabolism, lipid metabolism, small molecule metabolism, cholesterol biosynthesis, purine metabolism, and organophosphate metabolism (Figure 6). These differences in transporter expression may help explain the greater retention of PFOS in the female hCYP2B6-Tg mice. 

**Figure 8:**
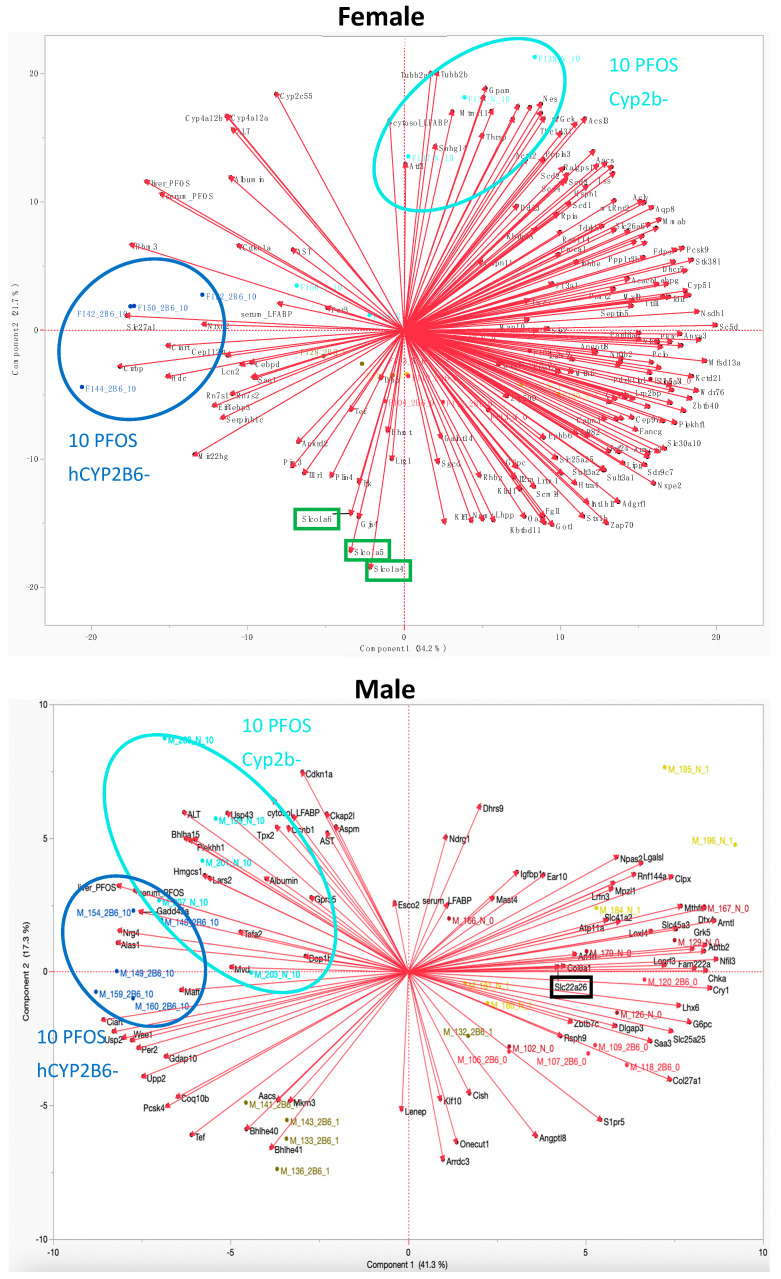
PCA of differentially expressed genes from Cyp2b-null and hCYP2B6-Tg female and male mice in comparison to serum and liver L-FABP, PFOS retention, and serum albumin, ALT, ALP, and AST.

PCA was performed on the RNAseq data comparing 10-PFOS-treated female and male Cyp2b-null and hCYP2B6-Tg mice to further decipher the putative reasons for differential toxicity and retention between the models (Figure 8). The PCA profiles of Cyp2b-null and hCYP2B6-Tg mice do not overlap for females, but overlap significantly in males, indicating greater differences between the mouse models in females. This is consistent with overt toxicity, number of gene expression changes, and PFOS retention. 

Changes in the expression of several genes between the two mouse models in females help explain differences in PFOS retention and toxicity. Most importantly, *oatp1a4* (*slco1a4*), *oatp1a5* (*slco1a5*), and *oatp1a6* (*slco1a6*) cluster together and are inversely associated with PFOS treatment in female Cyp2b-null mice. They are decreased in expression in Cyp2b-null mice by a log2 FC of −1.4, −1.4, and −1.6, respectively, compared to hCYP2B6-Tg female mice. Therefore, PCA suggests that we framed our hypothesis incorrectly, at least for these genes. We hypothesized that something in the hCYP2B6-Tg mice was altered that increased retention, when instead it appears that there are genes downregulated in the Cyp2b-null mice that reduced retention, probably through reduced uptake. Cyp2b-null mice also show an increase in *slc10a2* (asbt) relative to hCYP2B6-Tg mice treated with PFOS. In addition, Slc27a1 or *fatp1* (fatty acid transport protein 1), a long chain fatty acid transport protein, is increased in the hCYP2B6-Tg mice and is strongly associated with increased retention and, to a lesser extent, toxicity. 

Few transporters were disrupted in males. Slc22a26, an organic cation transporter (Oct22a26), was repressed (Figure 7). Other transporters that are differentially expressed between the 10-PFOS exposed male Cyp2b-null and hCYP2B6-Tg mice are slc4a2 and slc25a25, both Mg+ transporters. In general, the Cyp2b-null and hCYP2B6-Tg mice responded more alike and had overlapping PCA profiles. Differences between retention and differences in gene expression were both much smaller than females (Table 2). 

Several other genes were also perturbed in females (Figure 7). Transcription factors controlled by *Clock* and associated with diabetes, such as *Dba* and *Tef*, were downregulated in the Cyp2b-null mice. Genes involved in obesity or diabetes were also inversely or directly associated with treated Cyp2b-null mice, including Glycerol-3-phosphate acyltransferase 1 (*gpam*) and Perilipin-4 (*plin4*), which are involved in adipose and glycerolipid production [48]. Genes and parameters associated with toxicity (ALT, AST) include serum-fatty acid binding protein (*fabp1*; liver-fabp), *rbm3*, *per3*, *fatp1*, *cdkn1a*, *cyp4a12a*, *cyp4a12b*, and *cyp2c55*; and genes regulated by PPARs, PXR, and Clock [49,50,51,52]. 

Several PPARα/γ regulated genes are associated with retention and toxicity in the males based on the PCA (Figure 7). These include *cdkn1a*, *lars2*, *gadd45a*, *alas1*, *hmgcs1*, and *nrg4* [49,53,54,55,56], and *gpr55* is a PUFA-cannabinoid receptor [57]. This provides further evidence for the role of PPARs in the toxicity of PFOS [38,58,59]. 

### 3.5. Organic Anion Transport Protein Inhibitors Reduce PFOS Uptake

**Figure 9:**
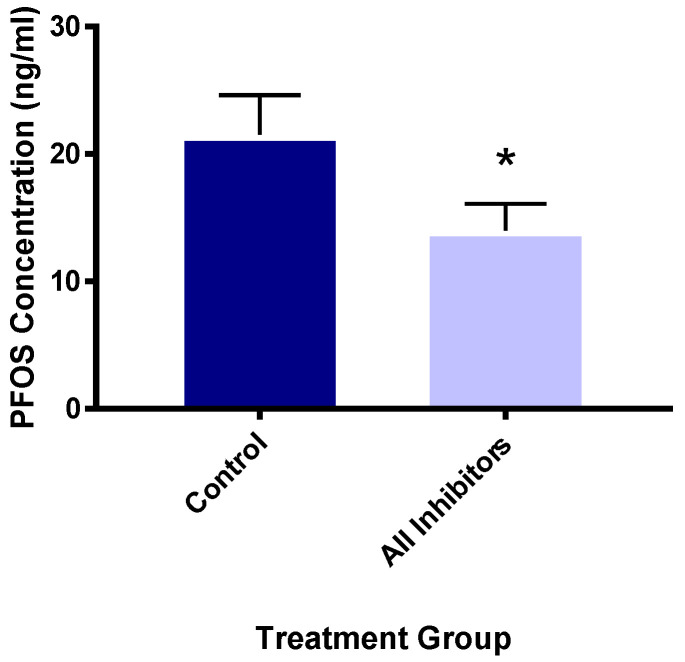
Oatp inhibitors reduce hepatocyte PFOS concentrations. PFOS concentrations (ng/mL) after incubation with 6 μM PFOS, 10 μM digoxin, 20 μM naringin, and 30 μM bromsulfthalein in primary murine hepatocytes. Data shown as mean ± SEM (n = 4–7). * indicates significance (*p* < 0.05) using Student’s *t*-test (GraphPad Prism 7.0).

Because of the greater retention differences in female mice, Oatp inhibitors were provided to female murine hepatocytes to test whether PFOS absorption could be decreased. Because Oatp1a4 was the most highly expressed transporter of the three, we targeted it with a relatively specific inhibitor, digoxin (10 μM) [60]. We used a mixture of 10 μM digoxin, 20 μM naringin, an Oatp1a5 inhibitor [61], and 30 μM bromsulfthalein, a broad Oatp inhibitor [62], to ensure all three Oatp1a members were inhibited. Compared to the no-inhibitor controls, the digoxin-only and inhibitor mixture groups showed 32.5 and 35.1% less PFOS retention (uptake). The digoxin and inhibitors/mixture treatments had very similar levels of PFOS, suggesting that digoxin was the main inhibitor of PFOS uptake with naringin and bromsulfthalein contributing little. Therefore, these groups were combined for statistical analysis. The inhibitor treatment group had significantly lower PFOS uptake and retention than the control hepatocytes (Figure 9), indicating that Oatp1a members transport PFOS. Earlier studies showed that rat OATP1A5 transports PFOS [32]. Murine Oatps have not been previously investigated for PFOS transport to our knowledge. These data suggest that murine Oatp1a4 likely transports PFOS. 

## 4. Discussion

hCYP2B6-Tg mice retain more PFOS than Cyp2b-null mice (Table 1), and for the most part this led to greater toxicity, especially in females. The reasons for increased PFOS retention in female and male hCYP2B6-Tg mice and the lower retention in female Cyp2b-null mice than male Cyp2b-null mice are unknown [10]. Therefore, the Cyp2b-null and hCYP2B6-Tg mouse models provide an unexpected and new system for studying this differential PFOS retention and toxicity in individuals. PFOS toxicity is associated with retention with a couple of exceptions: hCYP2B6-Tg have less steatosis at 1-PFOS and male hCYP2B6-Tg show slightly lower ALT levels at 10-PFOS despite the higher PFOS concentrations. Most other toxicity parameters follow PFOS retention, including PFOS-induced mortality in the hCYP2B6-Tg females [10]. 

Genes differentially expressed between the genotypes at each PFOS treatment were correlated with markers of liver toxicity and measured liver and serum PFOS concentrations via PCA. Female Cyp2b-null and hCYP2B6-Tg genes from 10-PFOS-treated mice clustered separately (Figure 8; Supplementary Materials). Three transporters (*oatp1a4*/*1a5*/*1a6*) were repressed in the female Cyp2b-null mice and found directly opposite to the 10-PFOS/Cyp2b-null cluster (Figure 8). This indicates that instead of hCYP2B6-Tg mice responding poorly, the Cyp2b-null mice responded well; they downregulated genes that can reduce retention. The OATPs are key uptake transporters in several tissues, including the liver and brain, with Oatp1a4 > Oatp1a6 showing the highest hepatic expression. OATPs are involved in the uptake of various organic anionic substrates from liver sinusoids, including exogenous drugs, such as statins and cardiac glycosides, as well as endogenous ligands, such as bile acids [63,64]. *Oatp1a4* is also female predominant [65]. Therefore, downregulation of these genes and in particular *oatp1a4* could reduce PFOS tissue absorption and concentrations in female Cyp2b-null mice (Table 1). 

These murine OATPs are orthologous to the human transporter, OATP1A2 [64,66]. Perfluorooctanoate (PFOA) is a known inhibitor of OATP1A2 transport, but does not transport perfluorooctanoate (PFOA) [67]. However, transport of PFOS by OATP1A2 has not been studied [68]. Additionally, Zhao et al., [32] demonstrated PFOS transport by rat Oatp1a5, as well as several other rat Oatps, including Oatp2b1, Oatp1b2, and Oatp1a1 [32]. Oatp1a1 and 1a5 are also orthologous to human OATP1A2 [64,66]. Rat OATP1A4 was not a PFOS substrate and the role of murine Oatp1a6 in PFOS uptake has not yet been studied [32]. We propose that the reduction of these Oatps in Cyp2b-null mice contributes to the decreased retention of PFOS in Cyp2b-null compared to hCYP2B6-Tg female mice. These transporters may also contribute to sexually dimorphic differences in uptake between male and female Cyp2b-null mice as they were more strongly regulated in females and *oatp1a4* is female predominant (Table 1; Figure 7; Supplementary Materials) [69]. 

Oatp inhibitors, digoxin, naringin, and bromsulfthalein, reduced PFOS retention (probably due to uptake) (Figure 8) in cultured murine female hepatocytes. with digoxin alone showing no difference (Supplementary Materials) from the mix, suggesting OATP1A4 plays a crucial role in PFOS uptake [60]. However, while digoxin is considered OATP1A4 specific, not all OATPs have been investigated and there may be crossover inhibition of different OATPs. Rat OATP1A5 was previously shown to transport PFOS [32], and therefore it is interesting to speculate that downregulation of this OATP that is primarily expressed in the intestine could reduce overall absorption into the body from food and water sources, in addition to a reduction in hepatic absorption [70]. 

Fatty acid transport protein 1 (*fatp1*; *slc27a1*), a long chain fatty acid transport protein, is increased in PFOS-treated hCYP2B6-Tg mice relative to Cyp2b-null mice in very close association with increased retention (Figure 8). It is possible that this transporter absorbs PFOS into the liver based on similar structure–function relationships, as PFOS looks like a fatty acid and binds to fatty acid binding proteins [71]. Therefore, PFOS-mediated increases in *fatp1* in hCYP2B6-Tg mice may lead to greater uptake of PFOS and fatty acids [10,34,72]. To our knowledge, PFOS transport by FATP1 has not been tested. Interestingly, *fatp1* is induced through PPARs and hCYP2B6-Tg mice show greater steatosis than Cyp2b-null mice [10,11], but only when provided a high-fat diet full of *fatp1* substrates. 

**Figure 10:**
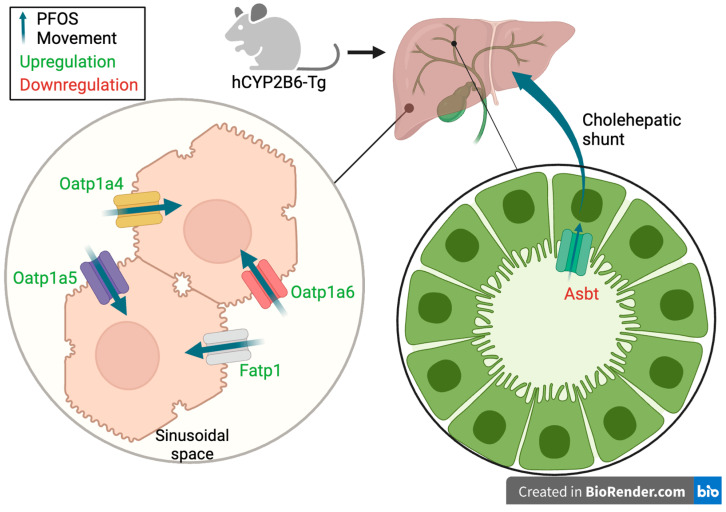
Schematic of transporter regulation in female hCYP2B6-Tg liver. Oatp1a4-6 and Fatp1, which are uptake transporters located along the basolateral membrane of hepatocytes, were upregulated (green) in hCYP2B6-Tg mice. We propose that this upregulation leads to greater PFOS uptake from the sinusoids into the hepatocytes, and thus greater overall PFOS retention in the liver of hCYP2B6-Tg mice consistent with differences in PFOS concentrations between Cyp2b-null and hCYP2B6-Tg mice. Asbt, a transporter located in cholangiocytes and involved in uptake from the bile duct, was downregulated (red). This would likely lead to less PFOS recycling via the cholehepatic shunt to hepatocytes.

*Asbt* (*slc10a2*), a bile acid transporter found along the cholangiocytes of the liver, is a known transporter of several PFASs from the bile ducts to the liver [32,63]. *Asbt* shows enrichment in Cyp2b-null mice relative to hCYP2B6-Tg mice treated with 10-PFOS. This is not consistent with decreased liver retention in Cyp2b-null mice. However, it may not be able to outcompete the changes in transport of fatp1 and the oatps. Furthermore, re-uptake of PFOS through the cholangiocytes may not be as common as sinusoidal entrance into the liver. Overall, our data suggest that Cyp2b-null mice retain less PFOS than hCYP2B6-Tg mice because of changes in the expression of several transporters, including, but maybe not limited to, FATP1 and several OATP1A members; however, unlike the OATPs and FATP1, the Asbt gene expression data are not consistent with this hypothesis (Figure 10). 

Few changes in transporter expression were observed in the males. In fact, male Cyp2b-null and hCYP2B6-Tg clusters following 10-PFOS (but not 1-PFOS; Suppl Materials) overlapped in their responses (Figure 7). In the male untreated, 1-, and 10-PFOS hCYP2B6-Tg groups, *Slc22a26* and *Slc25a25* were downregulated compared to their Cyp2b-null counterparts. Slc22a26 is a mouse-specific organic cation transporter [73], and Slc25a25 is a mitochondrial ATP-Mg^2+^/phosphate transporter typically regulated by circadian rhythm genes, such as CLOCK and BMAL1 [74]. Therefore, it is unlikely that either SLC22A26 or SLC25A25 are involved in PFOS uptake.

In addition to transporters, binding proteins, such as serum albumin and liver fatty acid binding protein (L-FABP), bind PFOS because of its structural similarity with their typical fatty acid ligands [68]. In females, serum L-FABP and albumin are associated with increased PFOS bioaccumulation and toxicity, but males did not show this same association. Thus, L-FABP and albumin sequestration of PFOS does not reduce toxicity in females and instead may lead to greater toxicity. 

PFOS exposure has previously been linked to perturbations in lipid metabolism and increased steatosis [7,10,34,40,75]. Our Chip Enrichment Factor Analysis (ChEA) via EnrichR showed that many groups had activation of PPARγ and PPARα pathways, indicating a mechanism by which PFOS induces changes in lipid metabolism. Others have also demonstrated that PFOS perturbs PPAR signaling and is associated with toxicity [10,38]. Both Cyp2b-null and hCYP2B6-Tg PFOS-treated mice experienced upregulation of GO biological processes associated with lipid metabolism, including the P450 epoxygenase pathway (*ephx1* and *ephx2*), which regulates the production of multiple dihydroxy-oxylipins from their epoxides, with hCYP2B6-Tg mice showing greater sensitivity to PFOS-mediated transcriptional effects (Table 2, Figure 6). In addition, biomarkers of PPAR signaling are associated with toxicity. For example, Cyp4a12a/b are highly associated with toxicity as measured with ALT in female hCYP2B6-Tg mice (Figure 7). Several CYPs in the CYP2-4 subfamilies also respond to steatosis and some may be protective [76]. These CYPs often produce 20-HETE (not measured) and 17,18-EpETE, which was significantly increased by PFOS (Figure 1 and Figure 2). Several other oxylipins were also increased by PFOS, including whole families of PUFA-derived oxylipins from EPA, AA, and DHA (Figure 1), and an increase in dihydroxy oxylipins that is correlated with an increase in epoxide hydrolase (Figure 2). 

Some oxylipins are also associated with reduced steatosis. For example, 19,20-EpDPA is decreased possibly due to the induction of epoxide hydrolases, and is lower in 1-PFOS-treated hCYP2B6-Tg that show less steatosis than Cyp2b-null mice (Figure 2). Previous research has shown that 19,20-EpDPA is inversely associated with hepatocyte lipid accumulation [12,77]. 9,10-DiHOME, which was induced by PFOS, stimulates adipogenesis through PPARγ [78]. PGE2 induces steatosis by inhibiting beta-oxidation and lipolysis [79] and was increased by PFOS, while inhibition of PGD2 production increases steatosis [80] and was decreased by PFOS. Each of these prostaglandins may provide insight as markers of PFOS hepatotoxicity or steatosis. 

Overall, there were few genotypic differences in oxylipin production (14,15-EpETE, 19,20-EpDPA); however, changes in oxylipins were sensitive to PFOS treatment (Figure 2 and Figure 3), with the prostaglandins showing the most sensitivity, as most were decreased and PGE2 was heavily increased. Interestingly, in the males, most of the metabolites that were inversely associated with PFOS retention and toxicity were DHA-derived (Figure 3A,D; Supplementary Materials). There was repression of total DHA content, free DHA species, and greater repression of epoxides, probably due to an increase in epoxide hydrolases. 

Unexpectedly, chow-diet-fed Cyp2b-null mice experienced increased steatosis compared to hCYP2B6-Tg mice with higher PFOS levels [10]. No GO terms were associated with increased steatosis in the Cyp2b-null mice, but several were associated with decreased steatosis in the hCYP2B6-Tg mice. Responses were sexually dimorphic, with females increasing lipid metabolism and transport and males increasing circadian rhythm genes. EnrichR (not shown; Supplementary Materials) suggests PPAR-regulated genes are involved, but also indicates Suz12 (female) and Clock (males) are involved. Interestingly, both of these transcription factors are circadian and sexually dimorphic [81,82]. Suz12 through its partner Ezh shows a stronger response in males and Clock in females, suggesting that, because of the constitutively lower responses, each response is stronger under PFOS duress. Chemicals flipping sex-dependent responses have been observed previously [83,84].

In general, toxicity is associated with PFOS retention, with the exception of steatosis in 1-PFOS-treated Cyp2b-null mice. We proposed earlier that the hCYP2B6-Tg mice may show a greater transcriptional response and thus be protected from steatosis. However, it is possible this steatosis was actually protective from overt PFOS toxicity by reducing free fatty acids through an increase in inert hepatic triglycerides. For example, steatosis can protect from non-esterified free fatty acid lipotoxicity, oxidative stress, and subsequent fibrosis [85]. Methionine and a choline-deficient-diet-induced non-alcoholic steatohepatitis progression may be slowed by increased triglycerides if inflammation and subsequent fibrosis are repressed [23]. Stroke patients with steatosis paradoxically have more favorable outcomes than those without steatosis [86], most likely by decreasing blood lipids. However, more research is needed on the initial reaction of the liver to steatotic agents to determine if steatosis is acutely protective but chronically toxic.

## 5. Conclusions

(1) We propose that differences in the retention of PFOS between Cyp2b-null and hCYP2B6-Tg mice may be regulated by the repression of uptake transporters, especially *oatp1a* family members in female Cyp2b-null mice. (2) hCYP2B6-Tg mice reacted differently to the PFOS treatment, with females enriching for lipid metabolism and organic anion transporters, and males enriching for circadian rhythm genes, providing for potential protective mechanisms from steatosis. (3) Regardless of the mouse model, changes in gene expression and liver toxicity parameters are often associated with PPARs and a few other transcription factors. 

## Figures and Tables

**Table 1:** Liver and serum PFOS concentrations as measured by LC-MS/MS in male and female Cyp2b-null and hCYP2B6-Tg mice treated with 0, 1, or 10 mg/kg/day PFOS for 21 days. Data are presented as mean ± SEM. Statistical significance was determined via one-way ANOVA on GraphPad Prism 7. “g” indicates a difference between genotypes at the same PFOS concentration, “p” indicates a difference between the control within a genotype, and “s” indicates a difference between sexes at a *p* < 0.01. Blue highlights differences due to PFOS concentrations and genotype; yellow highlights differences due to PFOS concentration, genotype, and sex.

Serum PFOS Concentration (ng/mL)						
**Treatment**	**Females**			**Males**		
PFOS (mg/kg/day)	0	1	10	0	1	10
Cyp2b-null	11.94 ± 0.89	65.70 ± 7.76 ^p^	312.90 ± 33.30 ^gps^	9.33 ± 0.99	86.20 ± 10.30 ^p^	408.10 ± 30.20 ^gps^
hCYP2B6-Tg	14.51 ± 0.63	101.00 ± 8.50 ^p^	545.70 ± 56.80 ^gp^	8.87 ± 0.71	96.70 ± 9.10 ^p^	593.80 ± 51.10 ^gp^
**Liver PFOS Concentration (** **μg/g tissue)**						
**Treatment**	**Females**			**Males**		
PFOS (mg/kg/day)	0	1	10	0	1	10
Cyp2b-null	28.41 ± 2.51	167.50 ± 8.88 ^p^	855.20 ± 25.60 ^gps^	27.53 ± 0.32	196.00 ± 9.10 ^p^	1013.00 ± 84.70 ^gps^
hCYP2B6-Tg	37.58 ± 3.71	235.50 ± 15.20 ^p^	1189.00 ± 46.70 ^gp^	31.72 ± 1.94	287.70 ± 25.90 ^p^	1156.00 ± 59.50 ^gp^

**Table 2:** Number of differentially expressed genes from the different treatment groups compared to appropriate controls ^a^.

	Differentially Expressed Genes in Females			Differentially Expressed Genes in Males		
Comparison	Upregulated genes	Downregulated genes	Total number of differentially expressed genes	Upregulated genes	Downregulated genes	Total number of differentially expressed genes
Cyp2b-null 0 vs. 1	24	12	36	82	28	110
Cyp2b-null 0 vs. 10	593	835	1428	594	781	1375
hCYP2B6-Tg 0 vs. 1	73	23	96	213	91	304
hCYP2B6-Tg 0 vs. 10	786	1095	1881	741	739	1480
Cyp2b-null vs. hCYP2B6 0 PFOS	4	2	6	3	10	13
Cyp2b-null vs. hCYP2B6 1 PFOS	3	4	7	31	43	74
Cyp2b-null vs. hCYP2B6 10 PFOS	75	160	235	6	12	18

^a^ Samples were either compared to untreated controls within a genotype or compared between similarly treated groups across the two genotypes (n = 4).

## Data Availability

The data from this study were deposited as Supplementary Materials in Mendeley Data, V1, doi: 10.17632/cjkhkn7fd4.3 and RNA sequencing data (RNAseq) was deposited in the Gene Expression Omnibus (GEO); GSE249617.

## Supplementary Materials

The following supporting information can be downloaded at: https://www.mdpi.com/article/10.3390/toxics12020106/s1, Supplementary Materials—all analyzed data—can be downloaded at: Mendeley Data, V3, doi: 10.17632/cjkhkn7fd4.3. (https://data.mendeley.com/datasets/cjkhkn7fd4/3) Access on 22 January 2024. RNA sequencing raw data (RNAseq) was deposited in the Gene Expression Omnibus (GEO); GSE249617.
